# Chitosan/poly(lactic-co-glycolic)acid Nanoparticle Formulations with Finely-Tuned Size Distributions for Enhanced Mucoadhesion

**DOI:** 10.3390/pharmaceutics14010095

**Published:** 2022-01-01

**Authors:** Feipeng Yang, Maleen Cabe, Hope A. Nowak, Kelly A. Langert

**Affiliations:** 1 Department of Molecular Pharmacology and Neuroscience, Loyola University Chicago Stritch School of Medicine, Maywood, IL 60153, USA; fyang26@hawk.iit.edu (F.Y.); mcabe1@luc.edu (M.C.); 2 Research Service, Edward Hines, Jr., VA Hospital, Hines, IL 60141, USA; nowakhop@grinnell.edu

**Keywords:** PLGA, nanoparticles, drug delivery, statin, size, surface functionalization, chitosan

## Abstract

Non-parenteral drug delivery systems using biomaterials have advantages over traditional parenteral strategies. For ocular and intranasal delivery, nanoparticulate systems must bind to and permeate through mucosal epithelium and other biological barriers. The incorporation of mucoadhesive and permeation-enhancing biomaterials such as chitosan facilitate this, but tend to increase the size and polydispersity of the nanoparticles, making practical optimization and implementation of mucoadhesive nanoparticle formulations a challenge. In this study, we adjusted key poly(lactic-co-glycolic) acid (PLGA) nanoparticle formulation parameters including the organic solvent and co-solvent, the concentration of polymer in the organic phase, the composition of the aqueous phase, the sonication amplitude, and the inclusion of chitosan in the aqueous phase. By doing so, we prepared four statistically unique size groups of PLGA NPs and equally-sized chitosan-PLGA NP counterparts. We loaded simvastatin, a candidate for novel ocular and intranasal delivery systems, into the nanoparticles to investigate the effects of size and surface modification on drug loading and release, and we quantified size- and surface-dependent changes in mucoadhesion in vitro. These methods and findings will contribute to the advancement of mucoadhesive nanoformulations for ocular and nose-to-brain drug delivery.

## 1. Introduction

Advances in biomaterials-based drug delivery systems (DDS) have expanded available options for non-parenteral drug administration, which bear advantages such as increased patient compliance and ease of administration over intramuscular, intrathecal, and intravenous injections [1,2]. Central to the success of non-parenteral delivery routes is the ability of a DDS to adhere to and permeate through mucosal epithelium. Chitosan (CS) [3] is a foundational mucoadhesive biomaterial that has been explored for non-parenteral administration routes including oral, topical, inhalation, nasal, and ocular [4,5,6,7,8]. CS is extracted from crustacean shells as its precursor chitin and subsequently deacetylated to varying degrees forming a linear polysaccharide of D-glycosamine [2,9]. Its chemical structure imparts a positive surface charge to particulate delivery systems [10], facilitating interactions with negatively-charged mucin through ionic, hydrogen, and hydrophobic interactions [11].

Size distribution of chitosan nanoformulations can vary from nanoscale [12,13] to microns [14,15], depending on synthesis protocols. For effective mucosal delivery, size in the nanoparticle (NP) range is an important factor. High surface area to volume ratio of small particles facilitates interfacial interactions with mucin as well as a diameter that allows for penetration of mesh spaces and channels within mucin matrix [2,16]. The structure of mucin meshes and viscosity of mucus fluids vary greatly throughout the human body [17] and depend on homeostatic or pathological conditions [18], which highlights the importance of refinement of CS NP size for the specific delivery route and application. Inclusion of chitosan in a given nanoformulation tends to increase the size and polydispersity (PDI) [19,20,21]. For ocular drug delivery, in addition to the above size requirements, particles in the nano-sized range are ideal for avoidance of discomfort, irritation, and foreign body sensation [22,23,24]. Intranasal administration is an emerging alternative for access to the central nervous system via uptake by the olfactory nerve after penetration of the nasal mucosal epithelium [2,25]. In this case, a size restriction of 100–300 nm has been identified [26,27].

For many pharmaceutical agents of interest, incorporation into and controlled release from chitosan-containing drug delivery systems is facilitated by the inclusion of a hydrophobic co-polymer such as poly(lactic-co-glycolic)acid (PLGA) [28]. Like chitosan, PLGA is biodegradable, biocompatible, and non-toxic. Controlled, local release from PLGA nanoparticles allows therapeutic doses to be achieved in the tissue of interest while minimizing systemic side effects and toxicity [29,30]. For example, statins, inhibitors of HMG-CoA reductase, have been demonstrated to exhibit beneficial pleiotropic effects [31] but high systemic doses are associated with myopathy and rhabdomyolysis [32]. We and others have recently shown that administration of statin-loaded PLGA nanoparticles modulates local immune and inflammatory responses [33,34]. Localized statin therapy has been investigated for ocular and nose-to-brain applications [28,34,35,36,37].

While NP size is an important parameter for mucosal delivery, the relationship between particle size and drug loading is complex, and the scientific principles that result in reduced size tend to be associated with lower drug loading [38,39]. Thus, formulation parameters must take into account not only optimal size, but sufficient encapsulation efficiency. In this study, we examined formulation parameters that result in the ability to finely tune the size and PDI of CS-PLGA nanoparticles loaded with simvastatin. We investigated drug encapsulation, drug release and the degree to which each formulation adhered to mucosal epithelial cells. Our protocol modifications resulted in four unique size groups of PLGA NPs and equally-sized CS-PLGA NP counterparts that demonstrated size- and surface-dependent differences in drug loading and mucoadhesion.

## 2. Materials and Methods

### 2.1. Materials

Ester-terminated poly(lactic-co-glycolic)acid (PLGA 85:15) was obtained from Lactel (Birmingham, AL, USA). Dichloromethane (DCM), acetonitrile (MeCN), dimethyl sulfoxide (DMSO), poly(vinyl alcohol) (PVA, 31,000–50,000 Da, 87–89% hydrolyzed), chitosan (low molecular weight (50,000–190,000 Da), 75–85% deacetylated) were purchased from Sigma-Aldrich (St. Louis, MO, USA). Simvastatin was obtained from Cayman Chemical (Ann Arbor, MI, USA). Fetal bovine serum (FBS), penicillin-streptomycin, amphotericin B were from Life Technologies (Grand Island, NY, USA), 1,1’-dioctadecyl-3,3,3’,3’-tetramethylindodicarbocyanine, 4-chlorobenzenesulfonate salt (DiD) was from Thermofisher Scientific (Waltham, MA, USA) and cOmpleteTM protease inhibitor cocktail was obtained from Roche (Basel, Switzerland).

### 2.2. Nanoparticle Synthesis

PLGA NPs were prepared by oil-in-water single emulsion, using commercially available polymer (85:15, viscosity 0.55–0.75, LACTEL Absorbable Polymers, Birmingham, AL, USA). PLGA polymer was first dissolved in an organic solvent (DCM ± MeCN, ratios indicated in Table 1) and added dropwise into an aqueous solution containing poly(vinyl alcohol) as a steric stabilizer (PVA, volume and concentration indicated in Table 1) under vigorous vortexing. The emulsion was formed by sonication using an ultrasonic processor (GE130PB, Cole-Parmer, Vernon Hills, IL, USA) for 10 rounds with 30 s on and 30 s off. After sonication, the emulsified mixture was added to a 1 L beaker containing PVA solution (the “evaporation condition” indicated in Table 1) and stirred overnight to allow the organic solvent to fully evaporate. The NPs were collected and washed with diH_2_O using Sorvall RC-5B refrigerated superspeed centrifuge (Thermo Fisher Scientific, Waltham, MA, USA). The NPs were then dispersed in 2% sucrose in diH_2_O, frozen at −80 °C, lyophilized overnight using Edwards K4 Modulyo Freeze Dryer (Thermo Fisher Scientific, Waltham, MA, USA), and stored at −20 °C in a desiccator. NP size was tuned by adjusting the ratio of MeCN cosolvent, the concentration of PLGA in the organic solvent, the composition of the aqueous phase, and the sonication amplitude (Table 1). Simvastatin (4% *w*/*w*, Cayman Chemical, Ann Arbor, MI, USA) and lipophilic tracer DiD (0.3% *w*/*w*, Invitrogen) were encapsulated into the NPs by addition to the organic phase.

Positively-charged, chitosan containing NPs were formed using the same protocol above but with the inclusion of chitosan in the PVA for emulsifying and evaporating at a final concentration of 0.5% *w*/*v*. Chitosan powder was dissolved in 1% acetic acid to form a 1% (*w*/*v*) chitosan stock solution and diluted 1:1 with PVA to make solutions with the final volumes and concentrations of PVA indicated in Table 1.

### 2.3. Nanoparticle Characterization

The sizes, PDI, and zeta potential of NPs were assessed using Zetasizer Nano ZS90 (Malvern Panalytical, Westborough, MA, USA) as described in our previous study [40]. Briefly, for size and PDI measurement, NPs were first suspended in diH_2_O at 0.1 mg/mL and equilibrated for 3 min in disposable polystyrene cuvette (DTS0012, Malvern Panalytical). The final measurement was the average of four separate and consecutive measurements. Similarly, for zeta potential measurement, NPs (0.1 mg/mL) were equilibrated for 1 min in folded capillary zeta cells (DTS1070, Malvern Panalytical) before being measured four times and taken the average as the final zeta measurement.

For transmission electron microscopy (TEM) imaging, carbon-coated 200 mesh copper grids (Electron Microscopy Sciences, Hatfield, PA, USA), pretreated with 0.002% Alcian blue in 0.03% acetic acid to increase the hydrophilicity of the grids, were floated on top of 30 µL drops of NP samples for 30 min at room temperature. After washing with diH_2_O, the sample was negatively stained by floating the grid on a 50 µL drop of filtered 2% uranyl acetate (pH 7) for 5 min at room temperature. The sample was placed into a grid storage box and allowed to dry for 12 h before imaging with a Philips CM120 transmission electron microscope (TSS Microscopy, Beaverton, OR, USA).

### 2.4. Drug Loading and In Vitro Release

To assess the drug loading of each preparation, a 4 mg/mL suspension was prepared in DMSO and sonicated for 5 min (Fisherbrand Model 505 Sonic Dismembrator with cup horn attachment (Qsonica LLC, Newtown, CT, USA)) to fully dissolve the polymer and extract the compound from the chitosan. Drug loading was quantified with Hitachi Elite LaChrom high-performance liquid chromatography (HPLC). Compounds were separated on a reversed-phase C18 column with isocratic elution of a mobile phase consisting of 70:30 acetonitrile: 0.05 N formic acid (*v*/*v*). Simvastatin was detected spectrophotometrically with an UV detector at 240 nm. Peak areas were integrated and compared to an external standard curve prepared from serial dilutions of a 4 mg/mL simvastatin stock in DMSO.

Release of simvastatin was determined using Slide-a-Lyzer MINI dialysis microtubes (69550, Thermo Scientific, molecular weight cut-off 3.5 kDa). 100 µL of a 4 mg/mL suspension of nanoparticles was added to each microtube, which was dialyzed into 1 L of phosphate buffered saline (PBS, pH 7.4) at 37 °C with gentle agitation. PBS was changed every 24 h to maintain sink conditions [30]. At each time point, microtubes were removed from the dialysate and the nanoparticle suspension was collected. Suspensions were dried with a Speedvac Vacuum Concentrator (Thermo Fisher Scientific, Waltham, MA, USA) and reconstituted in 80 µL DMSO. The remaining simvastatin was quantified with HPLC as described above.

### 2.5. Cell Culture

Human colon cancer HT-29 cells (ATCC) were maintained in McCoy’s 5A Medium (ATCC, Cat: 30-2007) supplemented with 10% FBS, and penicillin/streptomycin. The medium was changed every 2–3 days. Cells were used between passages 2–15.

### 2.6. Proliferation Assay

Cytotoxic effect of the nanoparticles on proliferation was evaluated using CellTiter 96^®^ Aqueous Non-Radioactive Cell Proliferation Assay (G5430, Promega, Madison, WI, USA) according to the manufacturer’s instructions. HT29 cells in 96-well plates were given serum-free media containing 0.2 mg/mL NPs for 1 h, rinsed with PBS, and subsequently cultured for 3 days in a regular medium. A combination of 100 µL fresh media and 20 µL of the proliferation assay reagent solution was added to each well and incubated for 1 h. The absorbance at 490 nm was measured using Cytation 5 Cell Imaging plate reader (Agilent Technologies, Santa Clara, CA, USA) and analyzed using GraphPad Prism 9.0.1 software.

### 2.7. Flow Cytometry

For flow cytometry analysis, HT-29 cells were treated with media containing different NPs (0.2 mg/mL, 0–2 h) and subsequently washed 3× with PBS. Cells were trypsinized, fixed with Image-iT™ fixative solution (FB002, Invitrogen, Waltham, MA, USA), and washed 3× with PBS. The suspended cells were measured using BD FACSCanto II Flow Cytometer (BD Biosciences, San Jose, CA, USA), and the results were analyzed using FlowJo software v10.7.

### 2.8. Statistics

One-way or two-way repeated-measures analysis of variance (ANOVA), followed with Tukey’s or Dunnett’s post hoc analysis, were used for comparison of data groups. In all cases, *p* < 0.05 was considered statistically significant.

## 3. Results

### 3.1. Nanoparticles with Different Size and Surface Functionalization

**Figure 1:**
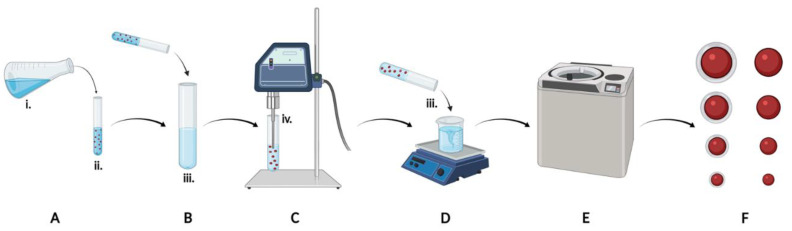
Graphical schematic of experimental design. Organic solvent (**A**) containing PLGA and simvastatin was added to aqueous emulsifying solution (**B**) containing PVA ± chitosan, sonicated (**C**), and poured into aqueous evaporating solution (**D**) containing PVA ± chitosan for removal of organic solvent overnight. NPs were collected by centrifugation (**E**), lyophilized, and eight formulations were characterized (**F**). Key formulation parameters included the organic solvent and co-solvent ratio (i), the concentration of polymer in the organic phase (ii), the composition of the aqueous phases (iii), and the sonication amplitude (iv).

**Figure 2:**
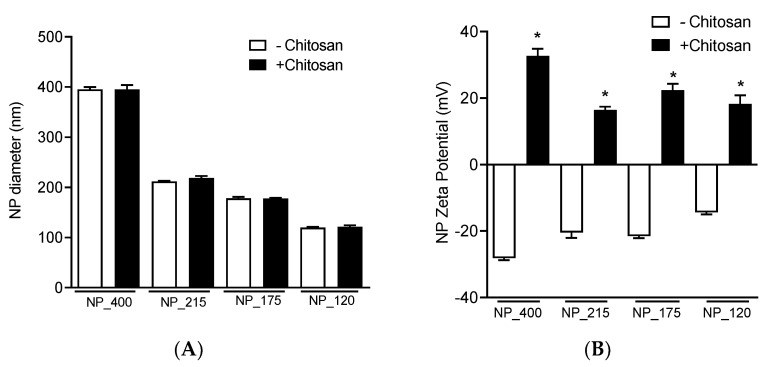

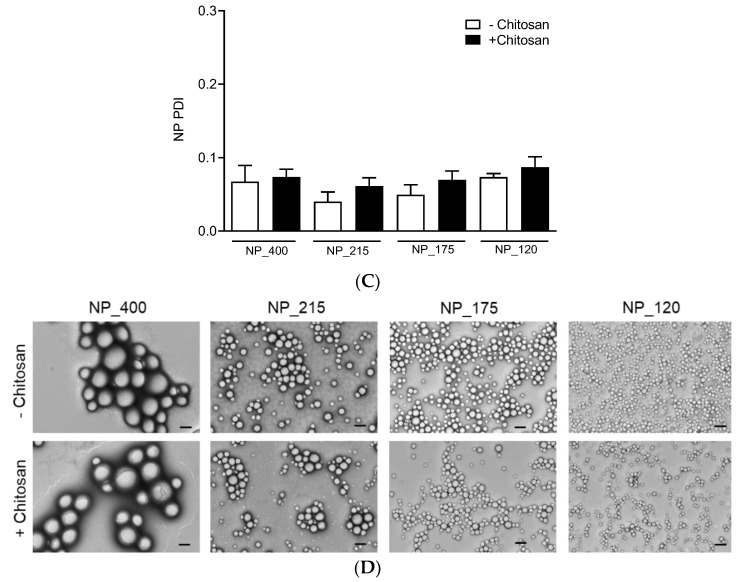
Nanoparticle characterization. Eight NP formulations were optimized to synthesize four statistically unique size groups of PLGA NPs (white bars) and equally-sized chitosan-containing controls (black bars). (**A**) Diameters (nm), (**B**) zeta potentials (mV), and (**C**) polydispersity indices (PDI) of the eight NP formulations. (**D**) TEM images of the eight NP formulations. Scale bar: 200 nm. For all datasets, values are means ± SD, *n* = 4 separately prepared lots. * *p* < 0.001 vs -Chitosan counterpart.

We optimized eight different nanoparticle (NP) formulations (Table 1, Figure 1) to establish four statistically unique size groups of PLGA NPs (NP_400^−^, NP_215^−^, NP_175^−^, NP_120^−^) and chitosan-functionalized counterparts (NP_400^+^, NP_215^+^, NP_175^+^, NP_120^+^). Within each size group, the size of PLGA NPs without and with chitosan functionalization were not statistically different from each other (Figure 2A). The incorporation of chitosan into the aqueous phase prior to emulsification resulted in the zeta potential of each sized formulation to switch from negative to positive (Figure 2B). The inclusion of chitosan in each nanoformulation did not significantly alter the PDI of the NPs (Figure 2C), which ranged from 0.040–0.087 (Table 2). Increasing PLGA concentration in the organic solvent (25–150 mg/mL) resulted in larger NP sizes, and including MeCN in the organic solvent (40–62.5%) resulted in smaller NP sizes (Table 1, Figure 2A). Representative TEM images for each NP group demonstrating monodisperse size distribution are shown in Figure 2D. 

### 3.2. Simvastatin Encapsulation and Release from the NPs

**Figure 3:**
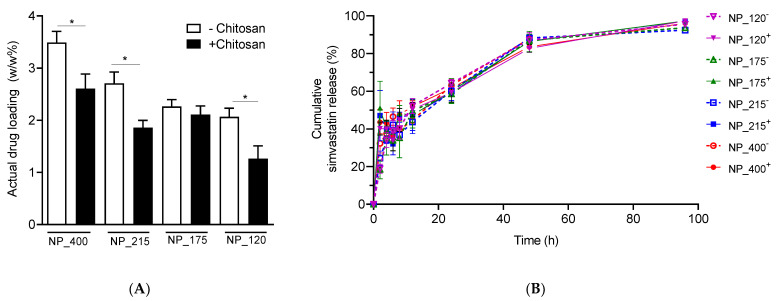
Simvastatin encapsulation and release. (**A**) Actual drug loading of eight NP formulations. (**B**) Cumulative release of simvastatin from each nanoformulation, as indicated. For all datasets, values are means ± SD, *n* = 4 separately prepared lots. One-way ANOVA followed by Tukey’s multiple comparisons test was performed. * *p* < 0.05 between indicated groups.

Local administration of optimized simvastatin delivery systems to ocular or nasal mucosal epithelium has the potential to harness anti-inflammatory pleiotropic effects of statins while maintaining low systemic doses [28,34,35,36,37]. We incorporated simvastatin into each nanoformulation by dissolving it in the organic phase with PLGA for a theoretical drug loading of 4% weight by weight (*w*/*w*). Actual drug loading ranged from 1.26 ± 0.25% *w*/*w* to 3.49 ± 0.22% *w*/*w* (Figure 3A), and calculated encapsulation efficiencies (EE) were size- and chitosan- dependent (Table 2). For the size groups of NP_400, NP_215, NP_175, and NP_120, EE of chitosan-containing formulations were 65.19 ± 7.0%, 46.5 ± 3.43%, 52.69 ± 4.15%, and 31.56 ± 6.17% compared to 87.25 ± 5.39%, 67.69 ± 5.44%, 56.63 ± 3.3%, and 51.63 ± 4.13 for PLGA only counterparts. Thus, within each size pair except NP_175, EE of chitosan-containing formulations was significantly lower than for PLGA only (*p* < 0.05, Figure 3A).

Others have demonstrated that chitosan coating delays drug release kinetics compared to non-functionalized PLGA NPs [13,41], and larger NP size has been observed to increase drug release rate from PLGA NPs [42]. We used Slide-a-Lyzer mini dialysis cassettes to evaluate drug release kinetics from each formulation. In our previous study, we quantified statins in the dialysate, which were present in hydroxyacid form after release from PLGA NPs [33]. Here, to maximize the dialysate volume and maintain sink conditions, we extracted and quantified the amount of simvastatin remaining in the NPs at each time point. Each formulation exhibited burst release, with 46.67 ± 4.89% (with chitosan) and 37.94 ± 6.01% (without chitosan) of the payload released by 8 h (Figure 3B, inset). Inclusion of chitosan in the formulation did not significantly attenuate this burst release or delay release kinetics. Each formulation achieved near-complete release of 96.97 ± 0.87% (with chitosan) and 93.87 ± 1.76% (without chitosan) by 96 h (Figure 3B).

### 3.3. Effects of NP Size and Functionalization on HT-29 Cellular Association

**Figure 4:**
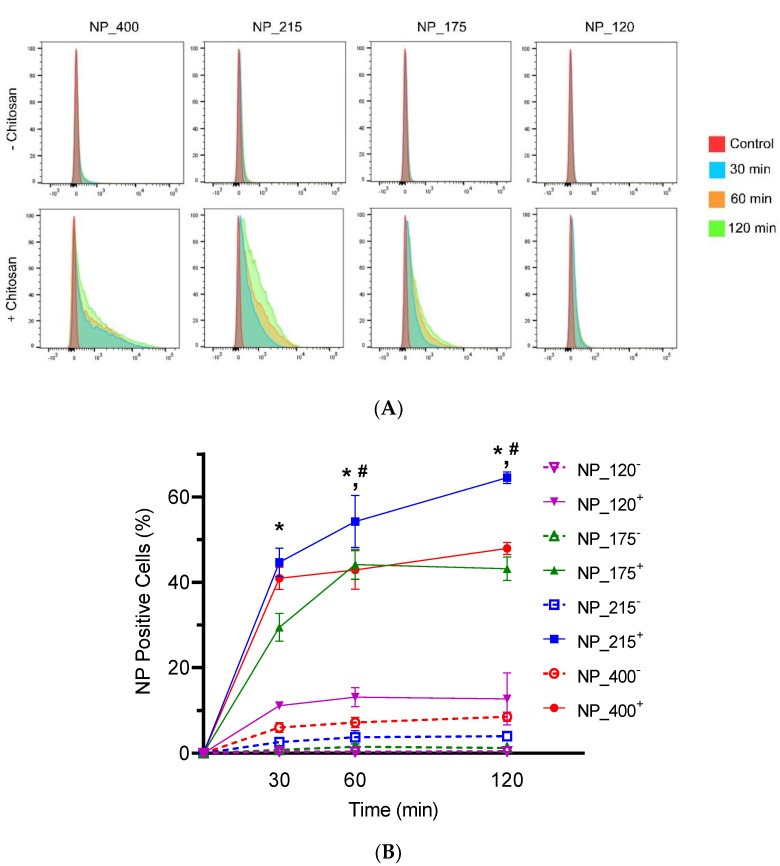
Adhesion of eight unique nanoformulations to mucosal epithelial cells. (**A**) Overlaid flow cytometry profiles of percent fluorescent cells at 30, 60, and 120 min for 400, 215, 175, and 120 nm PLGA NPs without (top panels) and with (bottom panels) chitosan, as indicated. (**B**) Quantification of percentage of NP positive cells at 30, 60, and 120 min. Data shown are mean ± SD, *n* = 4. * *p* < 0.05 for each NP^+^ vs. NP^−^ formulation of the same size at indicated time point; ^#^ *p* < 0.05 NP_215^+^ vs. all other conditions at indicated time point.

We evaluated the role of chitosan as a mucoadhesive biomaterial for each size of PLGA NPs. HT-29 mucosal epithelial cells were exposed to each nanoformulation for 30, 60, or 120 min, and flow cytometry was used to quantify the percentage of cells that retained NPs after washing. By using the percentage of NP-positive cells as the quantitative output, we eliminated size-dependent mean fluorescence intensity as a confounding variable. Within each size group, the inclusion of chitosan (solid traces) promoted significantly increased adhesion over negatively charged counterparts (dashed traces) at each time point (Figure 4A,B). Among the chitosan-containing nanoformulations, NP_120^+^ showed the lowest adhesion to HT-29 cells. At 60 and 120 min, NP_215^+^ exhibited significantly higher association than NP_400^+^, NP_175^+^, and NP_120^+^, and NP_215^+^ was the only nanoformulation to demonstrate an increase in adhesion from 60 min to 120 min (to 60 ± 4.5%).

### 3.4. Effect of Simvastatin Treatment on Cell Proliferation

**Figure 5:**
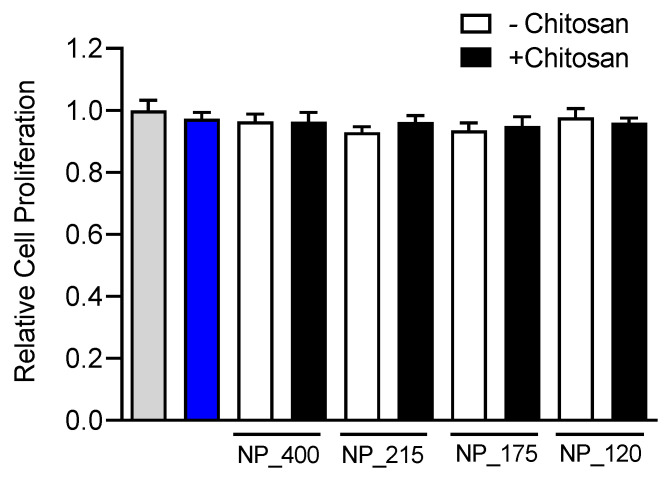
Effect of simvastatin treatment on cell proliferation. HT-29 mucosal epithelial cells were cultured in the presence of media alone (gray bar), 10 µM simvastatin, (blue bar), or control (white bars) or chitosan-containing (black bars) nanoformulations for 72 h. Proliferation was assessed with an MTS assay, and drug or NP induced changes in proliferation were compared to media-treated control. Values are means ± SD. *n* = 6, one-way ANOVA followed by Tukey’s multiple comparisons test.

Prior to investigating any novel nanoformulation in functional assays its potential for cytotoxicity must be evaluated. While chitosan has a favorable safety profile [43], a size dependent effect of chitosan nanoformulations on cytotoxicity has been observed [44]. Further, simvastatin has been demonstrated to significantly attenuate proliferation at doses above 10 µM in a cell-type dependent manner [45]. Thus, we evaluated the effect of free simvastatin or our nanoformulations on proliferation of HT-29 mucosal epithelial cells. Cells were cultured in the presence of free simvastatin (10 µM) or of a 10 µM equivalent amount of each simvastatin-loaded NP formulations for 1 h and subsequently cultured in culture media alone without drug or NPs for 3 days. There was no significant difference in the proliferation of HT-29 cells treated with each NP formulation or the free drug solution (Figure 5), indicating that the different formulations of NPs have no significant cytotoxic effect on the cells.

## 4. Discussion

Non-parenteral drug administration strategies bear several advantages over parenteral strategies including increased patient compliance and simple, outpatient dosing [2]. Despite these advantages, a requirement of non-parenteral delivery systems is that they overcome the mucosal epithelium and other biological barriers. Mucoadhesive and permeation-enhancing biomaterials can be incorporated into the formulation of non-parenteral delivery systems to increase drug delivery and efficacy. However, these materials tend to increase the size and polydispersity of nanoparticulate delivery systems [46], and different sites for mucosal delivery exhibit unique size restrictions and requirements [23,37], making practical optimization and refinement of mucoadhesive nanoparticle formulations a challenge.

In this study we utilized two biocompatible, biodegradable polymers, chitosan and PLGA. We formed four unique size groups of NPs that contained a negatively charged formulation and a positively charged, chitosan-containing formulation, resulting in eight unique nanoformulations that we assessed for drug loading, release, and mucoadhesion. Each formulation contained simvastatin at a theoretical drug loading of 4% *w*/*w*. Nanoparticle size was tuned by adjusting the concentration of PLGA in the organic phase, the sonication amplitude, the different concentrations of PVA, the ratio of dichloromethane and acetonitrile in the organic phase, and the addition of chitosan to the emulsifying and evaporating solutions.

As others have demonstrated, increasing the PLGA concentration in the organic phase was associated with larger NP size [39,47,48,49]. A PLGA concentration of 150 mg/mL was used to produce NPs with ~400 nm diameters. In our experiments, higher than 150 mg/mL PLGA did not yield batches of NPs with reproducible size distributions, likely due to the high viscosity of the organic phase. Sonication amplitude was used between 30% and 70% for all the NPs. Within this range, higher amplitude resulted in smaller NPs due to the increased shear force that dispersed the emulsion droplets [39]. When amplitudes below 30% were tested the resulting NPs displayed a high PDI, suggesting that at lower amplitudes the suspension was not uniformly emulsified.

In our study, lower PVA concentration resulted in increased NP size. We used a PVA concentration of 0.5% to produce ~400 nm NP and a PVA concentration of 6% to produce ~120 nm NP. Our tests showed that a lower than 0.5% or higher than 6% PVA concentration resulted in increased PDI and decreased reproducibility. It is thought that the concentration of PVA can contribute to smaller particle size at higher concentrations due to stabilization of the large surface area of small particles [39]. In an opposing mechanism, increased PVA concentration can also contribute to larger particle sizes by increasing the viscosity of the aqueous phase and thus reducing the shear forces available for droplet dispersion [12]. These competing factors must be optimized along with other processing parameters.

Inclusion of a water-miscible co-solvent in the organic phase was another strategy that we employed to reduce NP size and PDI. Acetonitrile at a volume % of 37.5–60 with dichloromethane was used to produce NP size groups with ~175 nm and ~120 nm diameters. When combined with dichloromethane, acetonitrile diffuses rapidly into the aqueous phase during solvent removal, resulting in an emulsion-diffusion mechanism of particle formation in addition to the emulsion-evaporation achieved with DCM alone. This facilitates a decrease in surface tension within the emulsion droplets and a reduction in particle size [50].

We modified the surface charge by including chitosan in the emulsifying and evaporating solutions. An alternative approach would be to adsorb or conjugate chitosan to the surface of preformed PLGA NPs [51,52,53]. We found it more feasible to tune the size of each nanoformulation and to obtain equally sized negative and positively charged counterparts by incorporating chitosan into NP synthesis. Positively charged NPs had a higher affinity to HT29 cells compared to negatively charged NPs, which is consistent with previous studies [13]. We found the NPs with the largest diameter had the highest positive (NP_400+) or negative surface charge (NP_400^−^), but that NP_400+ did not display the highest degree of cellular association (Figure 4). This suggests that surface charge alone does not determine mucoadhesion and provides further rationale for fine-tuning of CS-PLGA NP size.

We used simvastatin as a model drug to study the encapsulation efficiency and release of a hydrophobic molecule in PLGA NPs with different sizes and surface functionalization. For NPs with 400 nm, 215 nm, and 120 nm diameters, chitosan-containing PLGA NPs had lower drug encapsulation than unmodified PLGA NP counterparts. Interestingly, there was no significant difference in terms of drug release profile for all the nanoparticle groups. Future studies will address drug-dependent functional effects, such as GTPase activation and chemokine release [54,55,56], in primary mucosal epithelial cells to determine whether differences in mucoadhesion result in differential functional effects of each size formulation.

Limitations of our study include the use of the tumor-derived HT-29 mucosal epithelial cell line. HT-29 are a robust mucosal epithelial cell line with a phenotype that includes the ability to form polarized adherent monolayers and secrete mucus [57]. However, malignant cells exhibit key differences from primary cells including their threshold for cytotoxicity and drug response profile [58]. Thus, our findings are best interpreted in the context of identifying the formulation parameters that allow for fine-tuning of the size of chitosan-containing nanoformulations while maintaining a positive surface charge and narrow PDI. We can predict, but not conclude, how these nanoformulations will behave within physiologically relevant systems that model the ocular surface or the nasal epithelium.

Mucosal epithelia throughout the body exhibit variable biophysical properties, and size restrictions within the same tissue can vary with pathological state [2,17,18]. Thus, fine-tuning of the size of particulate mucosal drug delivery systems is a critical step before advancement to in vivo or clinical investigation. Strategies employed herein to establish four statistically unique size groups of PLGA NPs and equally-sized CS-PLGA NP counterparts will ensure that such investigations are able to be performed with the appropriate controls. Ultimately, these findings will contribute to the advancement of CS-PLGA nanoformulations for ocular and nose-to-brain drug delivery systems.

## Figures and Tables

**Table 1:** Nanoparticle Synthesis Formulations.

Group	[PLGA] (mg/mL)	Solvent	% DCM	Emulsifying Solution	Sonication Amplitude	Evaporating Solution
NP_400^+^	150	DCM ^1^ (1 mL)	100	1% PVA ^3^ and 0.5% Chitosan (4 mL)	30%	1% PVA and 0.5% Chitosan (45 mL)
NP_215^+^	100	DCM (1 mL)	100	3% PVA and 0.5% Chitosan (6 mL)	60%	0.7% PVA and 0.5% Chitosan (45 mL)
NP_175^+^	100	MeCN ^2^ (0.6 mL) + DCM (0.4 mL)	40	5% PVA and 0.5% Chitosan (6 mL)	70%	0.5% PVA and 0.5% Chitosan (45 mL)
NP_120^+^	25	MeCN (1.25 mL) + DCM (0.75 mL)	37.5	5% PVA and 0.5% Chitosan (10 mL)	70%	0.1% PVA and 0.5% Chitosan (45 mL)
NP_400^−^	150	DCM (1 mL)	100	0.5% PVA (6 mL)	40%	1% PVA (60 mL)
NP_215^−^	100	DCM (1 mL)	100	3% PVA (6 mL)	60%	0.7% PVA (45 mL)
NP_175^−^	100	MeCN (0.4 mL) + DCM (0.6 mL)	60	3% PVA (6 mL)	60%	0.7% PVA (45 mL)
NP_120^−^	100	MeCN (0.5 mL) + DCM (0.5 mL)	50	6% PVA (5 mL)	60%	0.5% PVA (50 mL)

^1^ DCM: dichloromethane. ^2^ MeCN: acetonitrile. ^3^ PVA: polyvinyl alcohol.

**Table 2:** Summary of Nanoparticle Characteristics.

Group	Size (nm)	PDI ^1^	Zeta Potential (mV)	Encapsulation Efficiency ^2^ (%)
NP_400^+^	395.2 ± 8.5	0.073 ± 0.011	+32.66 ± 2.20	65.19 ± 7.0
NP_215^+^	218.5 ± 3.5	0.061 ± 0.012	+16.42 ± 0.96	46.5 ± 3.43
NP_175^+^	177.7 ± 1.6	0.070 ± 0.012	+22.38 ± 1.95	52.69 ± 4.15
NP_120^+^	121.7 ± 2.6	0.087 ± 0.014	+18.26 ± 2.58	31.56 ± 6.17
NP_400^−^	395.0 ± 5.0	0.067 ± 0.022	−28.15 ± 0.65	87.25 ± 5.39
NP_215^−^	211.9 ± 1.4	0.040 ± 0.013	−20.46 ± 1.62	67.69 ± 5.44
NP_175^−^	178.1 ± 2.9	0.049 ± 0.014	−21.56 ± 0.59	56.63 ± 3.3
NP_120^−^	119.6 ± 1.5	0.073 ± 0.005	−14.39 ± 0.67	51.63 ± 4.13

^1^ PDI: polydispersity index; ^2^ Encapsulation efficiency = $\frac{actual\;drug\;loading}{theoretical\;drug\;loading}\times 100\%$

## Data Availability

The data that support the findings of this study are available from the corresponding author, K.A.L., upon request.
